# The use of *spa* and phage typing for characterization of clinical isolates of methicillin-resistant *Staphylococcus aureus* in the University Clinical Center in Gdańsk, Poland

**DOI:** 10.1007/s12223-012-0148-z

**Published:** 2012-04-26

**Authors:** Katarzyna Wiśniewska, Anna Szewczyk, Lidia Piechowicz, Marek Bronk, Alfred Samet, Krystyna Świeć

**Affiliations:** 1 Department of Medical Microbiology, Medical University of Gdańsk, Do Studzienki 38, 80-227 Gdańsk, Poland; 2 Department of Clinical Microbiology, University Clinical Center in Gdańsk, Smoluchowskiego 18, 80-214 Gdańsk, Poland

## Abstract

The emergence of spa types and *spa*–clonal complexes (CC) among clinical methicillin-resistant *Staphylococcus aureus* isolates collected from the University Clinical Center in Gdańsk between 2008 and 2009 were investigated. Phage typing was used as the initial screening in the study. The basic set of phages and the additional set of phages were used. Most of the isolates (56 %) belonged to the phage group III. With the additional set of phages, eight types were found, with predominant one MR8 (50 %). Sixteen distinct *spa* types were observed. The most frequent were t003 (22 %), t151 (16 %), and t008 (12 %). The *spa* types were clustered into two *spa*-CC and eight singletons. The predominant CC010 (50 %) consisted of six types, with the most common t003 (36.7 %) and t151(26.7 %), and in 80 % was identified as staphylococcal chromosomal casette *mec* (SCC*mec*) type II. The second cluster has no founder (12 %) with only two *spa* types: t037 belonging to SCC*mec* type III and t029. In the most frequent singleton, *spa* type t008 alone was clustered in 12 % of the isolates. All singletons correspond to SCC*mec* type IV. The CC010 was distributed in most of the hospital wards, corresponded to Multilocus sequence typing type ST5/ST225 and was constantly present throughout the observed period. The isolates of CC010 generally belonged to the phage group III, and most of them (53.3 %) were resistant to erythromycin, clindamycin, and ciprofloxacin. The concordance between *spa*-clone and phage type was very high, but the same phage type MR8 was observed within different *spa t*ypes of the predominant clone.

## Introduction

Methicillin-resistant *Staphylococcus aureus* (MRSA) is a significant nosocomial pathogen. In Europe, only a small number of countries have succeeded in controlling MRSA (Tiemersma et al. 2004). Epidemiological monitoring is essential for limiting the occurrence and spread of epidemic clones within and between hospitals. In the study of strain origin, adequate typing of MRSA has become an important tool. Several methods have been used to distinguish MRSA strains (Tenover et al. 1994). The “gold standard” is pulsed-field gel electrophoresis (PFGE) (Murchan et al. 2003). However, while PFGE has excellent discriminatory power, it is labor-intensive and difficult to standardize among different laboratories (van Belkum et al. 1998). The method which has become increasingly popular during recent years is *spa* typing (Strommenger et al. 2008). It has been shown that *spa* typing, in contrast to PFGE, can be used to study both the molecular evolution as well as hospital outbreaks of MRSA (Tang et al. 2000). Moreover, the implementation of software algorithms for the grouping of related sequence types enables the classification of isolates into particular clonal lineages (Rupptisch et al. 2006). A good correlation between the clonal groupings of MRSA obtained by *spa* typing and those obtained by other methods has been described (Strommenger et al. 2008; Tang et al. 2000).

Phage typing has been used for epidemiological surveillance of *S. aureus* strains for many years (Blair and Williams 1961; Widemauwe et al. 2004). Despite of being an internationally recognized method, the large number of non-typable strains limits its use, particularly with respect to MRSA. Thus, for typing of MRSA, a selected set of ten phages has been recommended (Richardson et al. 1999). Our earlier studies, based on phage typing, hypothesized that the predominant epidemic MRSA strains were periodically disseminated in various hospitals in Gdańsk region (Wiśniewska et al. 2000; Wiśniewska et al. 2007). In the present study, we investigated the emergence and diversity of *spa*–clonal complexes among single-patient MRSA isolates obtained for phage typing from the University Clinical Center (UCC) in Gdańsk between 2008 and 2009. We used phage typing as the initial screening in this study. Furthermore, we compared the results with those of the previous study and provided more information about predominant clones.

## Materials and methods

### Bacterial isolates

The study was performed on 50 MRSA isolates from patients of 20 various wards or departments of the UCC including: surgery 18 %; dermatology, venerology, and allergology 12 %; neurology 12 %; outpatients 10 %; plastic surgery and burns care 8 %; and pneumology 6 %. The strains were isolated in the hospital laboratory during the period March 2008–March 2009 and were sent to the Microbiology Department of the Medical University of Gdańsk for typing. The first isolate per patient was included in the study. Bacteria were cultured from swabs: wound (28 %), nose (14 %), skin (6 %), bedsore (6 %), throat (4 %), anus (4 %); fluids: tracheostomy tube fluid (8 %), blood (6 %), abscess fluid, pus (6 %), sputum (4 %), BAL (4 %), bronchial fluid (4 %); and single isolates from eye, urine, and catheter. The isolates were collected from colonized or infected patients as the only one isolated bacteria. The data about clinical recognitions were not available. The isolates were cultured on sheep blood agar (Oxoid, UK) and finally identified as *S. aureus* by API ID32 Staph system (bio Mérieux, France). Resistance to methicillin was primary tested using disk diffusion method on Mueller Hinton agar (Oxoid, UK) with oxacillin end cefoxitin (Becton Dickinson, Germany) and confirmed by detection of *mec A* gene by the polymerase chain reaction (PCR) as described by Barski et al. 1996. DNA of bacterial isolates was extracted according to the previous report (Barski et al. 1996). For further analyses, the isolates were sub-cultured on nutrient broth and stored with glycerol at −70°C.

### *Spa* typing and BURP analyses

The polymorphic X region of the protein A gene (*spa*) was amplified from the isolates by PCR with primers and according to procedure described by Kobayashi et al. 1999. *Spa* types were determined by using Ridom Staph Type software according to Harmsen et al. 2003. The *spa* types were clustered into *spa*–clonal complexes (CCs) using the algorithm based upon repeat pattern (BURP) (Rupptisch et al. 2006).

### SCC*mec* typing

Typing of the staphylococcal chromosomal casette *mec* (SCC*mec*) was done as described previously (Milheiriço et al. 2007).

### Phage typing

Phage typing was performed according to the recommendation of Blair and Williams 1961. Two set of phages were used as follows: a basic set of 23 phages with additional phages 88, 89, and 187 and an additional set of phages MR8, MR12, MR25, 30, 33, 38, M3, M5, 622, and 56B supplied by the Central Public Health Laboratory in London for use on MRSA strains (Richardson et al. 1999). The phages were used in concentrations at routine test dilution (RTD) and 100 × RTD. The phage type was defined by all the phages with strong reaction.

## Antimicrobial drug susceptibility testing

The susceptibility of the MRSA isolates to antimicrobial agents was determined by the disk diffusion method according to the guidelines of CLSI 2006 and Hryniewicz et al. 2005. The following drugs were used for test: erythromycin, clindamycin, ciprofloxacin, co-trimoxazole, tetracycline, gentamicin, vancomycin, teicoplanin, fusidic acid, rifampicin, linezolid, quinupristin-dalfopristin, chloramphenicol (Becton Dickinson, Germany), and mupirocin (Oxoid, UK). For isolates identified as resistant to erythromycin, but susceptible to clindamycin, *D* test was performed to detect inducible clindamycin resistance (Fiebelkorn et al. 2003). For vancomycin, additionally, the minimal inhibitory concentration (MIC) was determined by *E* tests as described by the manufacturer (AB Biodisc, Sweden).

### MLST analysis

Multilocus sequence typing (MLST) was conducted as described previously (Enright et al. 2000) on samples for every spa-type within predominant spa-clonal complex. MLST sequence types (ST) were assigned through the MLST database (www.mlst.net).

## Results

According to the classification using the basic set of phages, most of the isolates (56 %) belonged to the phage group III (Table 1). Twelve percent of the isolates was in the phage group II, and none in group I. Mixed groups or inhibition reactions were observed occasionally, and 30 % of the isolates were resistant to phages of the basic set. With the additional set of phages, eight types were identified. In 50 %, it was phage type MR8, the type MR25 in 8 %, and the type 622 in 6 %. The remaining types were found occasionally. The non-typable isolates accounted 26 %.

**Table 1:** Results of phage typing of the 50 MRSA isolates

Phage group	Number (%) of isolates	Phage type (basic set)	Number (%) of isolates	Phage type (additional set)	Number (%) of isolates
II	3 (6)	55	2 (4)	MR8	25 (50)
		55/71	1 (2)	MR25	4 (8)
III	28 (56)	54/75/83A	10 (20)	622	3 (6)
		75/83A	5 (10)	MR8/MR12	1 (2)
		47/54/75/83A	3 (6)	M8/MR25	1 (2)
		83A	3 (6)	MR8/MR12/MR25	1 (2)
		53/83A	2 (4)	M3	1 (2)
		75	2 (4)	MR25°/M5°	1 (2)
		6	1 (2)	NT	13 (26)
		47/75	1 (2)		
		42E/53/75/83A	2 (4)		
Mixed group:	2 (4)				
I/II/III		52A/3 C/55/84	1 (2)		
I/V		55/94/96	1 (2)		
Inhibition	2 (4)	88 °/89 °	1 (2)		
		55 °	1 (2)		
Non-typable	15 (30)	NT	15 (30)		

Looking at the frequency of resistance to antimicrobial agents of the strains (Table 2) revealed that the resistances to ciprofloxacin, erythromycin, and clindamycin were most frequent (86, 76, and 84 %, respectively). Inducible resistance to clindamycin was limited to one isolate (not shown), 42 % were resistant to tetracycline and 32 % to chloramphenicol (14 % were resistant to both antibiotics; not shown). Resistance to gentamicin, co-trimoxazole, and rifampicin were found in 16, 10, and 2 %, respectively. All isolates were sensitive to vancomycin, teicoplanin, fusidic acid, linezolid, mupirocin, and quinupristin-dalfopristin. The MIC_s_ vancomycin ranged between 0.75 and 1 μg/mL (not shown).

**Table 2:** Frequency of resistance to antimicrobial agents among the 50 MRSA isolates

Antimicrobial agent:	E	Cc	Cip	Te	C	Sxt	Ge	Rf
Number of resistant strains (%)	38 (76)	37 (74)	43 (86)	21 (42)	16 (32)	5 (10)	8 (16)	1 (2)

*E* erythromycin, *Cc* clindamycin, *Cip* ciprofloxacin, *Te* tetracycline, *C* chloramphenicol, *Sxt* co-trimoxazole, *Ge* gentamicin, *Rf* rifampicin

Sixteen distinct *spa* types were observed (Table 3). The most frequent were t003 (22 %), t151 (16 %), and t008 (12 %). The remaining types accounted for between 10 and 2 % each. BURP analyses of the *spa* types identified two clusters and eight singletons. The predominant was CC010, which harbored 50 % of MRSA isolates. It consisted of the strains associated with six types: t003, t151, t002, t2065, t045, and t010. The most common within CC010 were t003 (11/30 = 36.7 %) and t151 (8/30 = 26.7 %). Most of the strains of CC010 (80 %) were identified as identical SCC*mec* type II with five bands of 162, 209, 284, 311, and 342 bp (the results of the PCR not shown). The remaining strains of three types of this complex harboring SCC*mec* type IV and were positive for 343 bp alone (t2065 and t010) or additionally for 311 bp (t045). The second cluster identified has no founder (12 % of the isolates). Only two *spa* types: t037 (all harboring SCC*mec* type III with 209 and 243 bp) and t029 (SCC*mec* IV type) were assigned to this cluster. Among eight singletons identified, the most frequent was represented by 12 % of the isolates and was consisted of only *spa* type t008. Six singletons were unique to one isolate. The strains from all singletons were identified as SCC*mec* type IV as to be positive for 311 bp alone or additionally for 343 bp.

**Table 3:** Results of *spa*-typing and *Spa*-complex analysis of 50 MRSA isolates in correlation with SCC*mec* type

*Spa-*complex	Number (%) of isolates	*spa*-type (number of isolates)	Scc*mec* type
CC 010	30 (60)	t 003 (11)	II
		t 151 (8)	II
		t 002 (5)	II
		t 2065 (3)	IV
		t 045 (2)	IV
		t 010 (1)	IV
No founder	6 (12)	t 037 (5)	III
		t 029 (1)	IV
Singleton #1	6 (12)	t 008 (6)	IV
Singleton #2	1 (2)	t 018 (1)	IV
Singleton #3	1 (2)	t 032 (1)	IV
Singleton #4	1 (2)	t 127 (1)	IV
Singleton #5	1 (2)	t 346 (1)	IV
Singleton #6	2 (4)	t 437 (2)	IV
Singleton #7	1 (2)	t 899 (1)	IV
Singleton #8	1 (2)	t 1328 (1)	IV

The isolates belonging to the predominant cluster CC010 was distributed in 13 wards or clinical departments (Table 4) with the most frequency in dermatology, venerology, and allergology department (6/30 = 20 %) and neurology ward (5/30 = 16.7 %). The prevalent cluster was disseminated in different clinical settings and was constantly present throughout the observed period. Most of the isolates of CC010 (16/30 = 53.3 %) were resistant to erythromycin, clindamycin, and ciprofloxacin, other twelve (40 %) were resistant to chloramphenicol and six (20 %) to tetracycline. The isolates resistant to tetracycline were sensitive to macrolides. Eighty-three percent (25/30) isolates of CC010 belonged to the phage group III. The most common phage types were 47/54/75/83A and 54/75/83A (12/30 = 40 %). Four of the isolates of CC010 were non-typable with basic set of phages (4/30 = 13.3 %), and one belonged to the phage group II. The additional set of phages was unable to type only three isolates (10 %), including two isolates non-typable with the basic set. Irrespective of *spa* type, the isolates belonged to the predominant type which was MR8 (23/30 = 80 %). The isolates non-typable by additional set of phages and three isolates identified as type 622, in contrast to phage type MR8, were sensitive to erythromycin and clindamycin, but generally resistant to ciprofloxacin, tetracycline, and chloramphenicol. According to MLST, two related sequence types were identified within CC10. The isolate representing the *spa* type t003 was classified as ST225 and the remaining ones as ST5 (not shown in the table).

**Table 4:** Characteristic of the 30 MRSA isolates belonging to spa-CC010 by the results of phage typing and antibiotic resistance patterns analysis

Isolate	Date of isolation	Ward/clinical department	Clinical specimen	Antibiotic resistance	Phage group	Phage type (basic set)	Phage type (additional)	*Spa* type
1	20.03	Plastic surgery	Burn wound	Cip Te C	III	6	622	t2065
2	12.03	Pediatric nefrology	Skin	Cip Te	NT	NT	NT	t045
3	18.05	DWA	Sputum	Cip Te C	III	75	NT	t2065
4	6.06	Neurology	Blood	E Cc Cip	III	75/83A	MR8	t003
5	11.06	DWA	Urine	Te C	III	42E/53/75/83A	622	t010
6	19.06	Neonatology	Eye	E /Cc Cip	III	83A	MR8	t002
7	20.08	Neurology	Nose	E Cc Cip	III	83A	MR8	t002
8	29.08	Pediatric nefrology	Catheter ostium	Cip Te	NT	NT	NT	t045
9	2.09	Pneumology	Wound	E Cc Cip	III	83A	MR8	t002
10	5.09	Pneumology	BAL	E Cc Cip C	III	54/75/83A	MR8	t151
11	6.09	DWA	Throat	E Cc Cip C	III	54/75/83A	MR8	t151
12	8.09	Neonatology	Anus	E Cc Cip C	III	54/75/83A	MR8	t151
13	9.09	DWA	Nose	E Cc Cip C	III	54/75/83A	MR8	t151
14	10.09	Neurology	Bronchial fluid	E Cc Cip	III	54/75/83A	MR8	t003
15	15.09	Cardiosurgery	Wound	E Cc Cip	III	54/75/83A	MR8	t003
16	19.09	ICU	Nose	E Cc Cip	III	54/75/83A	MR8	t003
17	22.09	Neurology	Tracheostomy tube	E Cc Cip	III	47/54/75/83A	MR8	t003
18	4.10	Neurology	Sputum	E Cc Cip	III	47/75	MR8	t003
19	12.10	No data	Abscess	E Cc Cip	III	75/83A	MR8	t003
20	28.10	Trauma surgery	Nose	E Cc Cip	III	75/83A	MR8	t003
21	28.10	DWA	Wound	Cip Te C	III	53/83A	622	t2065
22	13.11	Plastic surgery	Wound	E Cc Cip	II	55/71	MR8	t003
23	24.11	Plastic surgery	Bedsore	E Cc Cip	III	54/75/83A	MR8	t003
24	18.01	ICU	BAL	E Cc Cip C	III	75/83A	MR8	t151
25	27.01	Trauma surgery	Tracheostomy tube	E Cc Cip C	III	47/54/75/83A	MR8	t151
26	8.02	Neurosurgery	Wound	E Cc Cip	III	83A	MR8/MR12	t003
27	24.02	DWA	Nose	E Cc Cip C	III	54/75/83A	MR8	t151
28	17.03	Hematology	Anus	E Cc Cip C	III	54/75/83A	MR8	t151
29	31.03	Trauma surgery	Wound	E Cc Cip	NT	NT	MR8	t002
30	3.04	Trauma surgery	Wound	E Cc Cip	NT	NT	MR8	t002

*ICU* Intensive Care Unit, *DWA* dermatology, venerology, allergology/inducible resistance to macrolides

## Discussion

We used phage typing as the first step in monitoring the epidemiology of MRSA infection in the main clinical center in North of Poland. The initial Polish investigations with the additional set of phages were carried out on 150 clinical MRSA isolates collected by the Microbiology Department of the Medical University of Gdańsk between 1990 and 1998 (Piechowicz et al. 1999). In contrast to the results of the present study, the initial survey showed 96.7 % of the isolates as typable. Moreover, in 1997–1998, in most of hospitals of Gdańsk region, including the UCC, predominated the MRSA strains belonging to the new phage type MR25/M5, resistant to erythromycin, clindamycin, ciprofloxacin, doxycycline, gentamicin, and rifampicin and resistant or medium susceptible to fusidic acid (Wiśniewska et al. 2000). The study performed more recently in five selected hospitals in Gdańsk area showed that in 2005–2007, the phage type MR25/M5 disappeared but the most frequent was the type 56B, resistant to erythromycin, clindamycin, ciprofloxacin, tetracycline, gentamicin, and co-trimoxazole (Wiśniewska et al. 2007). The present study showed that a significant shift in the prevalence of phage types was achieved as over 10 years later; the most common phage type in the UCC was MR8. Moreover, if we accept the guideline of Blair and Williams 1961, that isolates may be considered to be different if they differed in strong reactions to more than one phage, we found only 6 % of typable isolates from this center to be quite different.

Our study confirmed the general trends that most of typable MRSA strains belong to the phage group III. However, compared to the previous study we performed, this study revealed a remarkable increase in the number of isolates non-typable by the additional set of phages (Piechowicz et al. 1999; Wiśniewska et al. 2007). With respect to the basic set of phages, a non-typability was comparably high. The survey performed on Belgian MRSA isolates collected from 93 different hospitals between 1992 and 2001 showed that typability of these strains by basic set of phages was at least 95 %, except for the year 1997 where 9 % of the studied population were non-typable, but it was mostly due to 26 % non-typability in one hospital (Widemauwe et al. 2004). In other countries, problems due to non-typable MRSA have also been encountered (Samra et al. 2001).

In our earlier studies with phage typing, no molecular data were included. Phage typing has been the classical method for detecting epidemic and pandemic strains, although confirmation of selected isolates by molecular methods is now needed. *Spa* typing appears to have significant advantages over many currently available methods. The main advantage is the portability of sequence data which greatly simplifies the sharing of information between laboratories and facilitates the creation of the large-scale database for the study of global as well as local epidemiology (Harmsen et al. 2003; Strommenger et al. 2008). The spa types among MRSA strains are area specific (Fenner et al. 2008; Fridrichova et al. 2010; Marchese et al. 2009). The ability of *spa* typing to distinguish strains makes it particularly well suited for initial screening that may be used for further identification of the strains (Rupptisch et al. 2006). Looking at sequence database for *spa* types, specific repeats seem to be associated with particular MLST sequence types. From the sixteen spa types observed in our study, six constituted one predominant clonal lineage with the most frequent t003/ST225. Moreover, all of the remaining *spa* types clustered into the CC010 belonged to the worldwide widespread MLST type ST5. *Spa* type 003 deposited in the *spa* server corresponds to the epidemic clone isolated in the USA and in many European countries, including the neighboring countries of Poland (Strommenger et al. 2008). The *spa* type t002, which in our study also belonged to the CC010, is the fourth most frequent *spa* type worldwide among MSSA and MRSA isolates (Fenner et al. 2008). Similarly, s*pa* type t008, in our study clustered in the most frequent singleton #1, belonged to the top ten *spa* types in this database. If only predominant *spa* CC010 data were analyzed, the concordance between *spa* clone and phage type was very high, but the dominant phage type MR8 was observed in the isolates belonging to the different *spa t*ypes. A promising fact is that only 10 % of the isolates of CC010 are non-typable by the additional set of phages. Thus, the high number of non-typable isolates observed in this study was caused by the presence of MRSA clusters which occasionally occurred in the center.

In comparison with the results of our previous study, in the present study, multi-resistant strains were found occasionally (Wiśniewska et al. 2000) and the most prevalent strains were resistant only to macrolides and ciprofloxacin. In other countries in Europe, a similar trend in levels of antibiotic resistance can be observed (Marchese et al. 2009; Kluytmans-Vandenbergh and Kluytmans 2006). Since the 1990s, community-associated MRSA (CA-MRSA) has emerged worldwide (Bassetti et al. 2009). This strains, unlike to hospital acquired MRSA (HA-MRSA) are generally more sensitive to non-β-lactam antibiotics (Naimi et al. 2003). The potential risk of its introduction into hospitals are currently matters of great concern. HA-MRSA and CA-MRSA can be distinguished on the basis of the phenotype and genetic profile in SCC*mec* typing (Naimi et al. 2003). SCC*mec* types I–III carry additional genes that provide resistance to antibiotics other than ß-lactams and are most often found in HA-MRSA. SCC*mec* types IV and V usually carry no additional drug resistance genes and are mainly associated with CA-MRSA. In our study, almost half of the MRSA population showed a pattern band characteristic for SCC*mec* type II. Moreover, this type was associated exclusively with the *spa*-clonal complex predominantly found in the hospital and corresponded with one of the most frequent hospital clone ST5 or its single-locus variant Rhine-Hesse clone ST225. Both of these STs recently have been observed as an epidemic hospital clones in Poland and in neighboring countries (Łuczak-Kadłubowska et al. 2008). SCC*mec* type III was sporadically occurring and related to the second cluster identified. As it was shown, irrespective of the *spa* type and *spa* clusters, that SCC*mec* type IV circulated in the hospital. The fact that the amplification patterns of such strains differed only in one band showed that they occurred in limited genetic variants of the *mec* cassette. Most of the strains of SCC*mec* type IV, including predominant *spa-*CC, as expected, were sensitive to more antibiotics than the strains belonging to SCC*mec* II and III. However, within other *spa* types, in single cases, resistance patterns were similar, especially with respect to fluoroquinolones, clindamycin, tetracyclines, and gentamicin. This observation suggests appearance of unique variant subtypes.

In conclusion, by this study, we demonstrate that phage typing is still an internationally recognized method and, with addition of *spa* typing, may be a useful tool for rapid research of correlation between MRSA isolates. This is the first attempt at using these methods in combination, with respect to MRSA population of one of the main clinical center in the North of Poland. Although, our investigation may be considered to be an initial screening for studies providing full genetic characteristic of MRSA strains isolated in the UCC. Further study should focus on monitoring of changes in the resistance profile of MRSA and search for emergence of CA-MRSA in this hospital. According to the report from a countrywide survey, MRSA clone, possibly representing community-acquired strain, are circulating in Polish hospitals (Łuczak-Kadłubowska et al. 2008). One of the distinguishing features of CA-MRSA is that high percentage of such strains are positive for Panton–Valentine leukocidin gene (Naimi et al. 2003). Thus, such investigation would be on interest.
